# Quantitative Proteomics Uncovers the Interaction between a Virulence Factor and Mutanobactin Synthetases in *Streptococcus mutans*

**DOI:** 10.1128/mSphere.00429-19

**Published:** 2019-09-25

**Authors:** Katherine Rainey, Landon Wilson, Stephen Barnes, Hui Wu

**Affiliations:** a Department of Microbiology, Schools of Dentistry and Medicine, University of Alabama at Birmingham, Birmingham, Alabama, USA; b Department of Pediatric Dentistry, Schools of Dentistry and Medicine, University of Alabama at Birmingham, Birmingham, Alabama, USA; c Department of Pharmacology and Toxicology, Schools of Dentistry and Medicine, University of Alabama at Birmingham, Birmingham, Alabama, USA; d Targeted Metabolomics and Proteomics Laboratory, Schools of Dentistry and Medicine, University of Alabama at Birmingham, Birmingham, Alabama, USA; University of Kentucky

**Keywords:** *Streptococcus mutans*, oxidative stress, mutacin, mutanobactin, proteomics, competition

## Abstract

Streptococcus mutans, the primary etiological agent of tooth decay, has developed multiple adhesion and virulence factors which enable it to colonize and compete with other bacteria. The putative glycosyltransferase SMU_833 is important for the virulence of S. mutans by altering the biofilm matrix composition and cariogenicity. In this study, we further characterized the *smu_833* mutant by evaluating its effects on bacterial fitness. Loss of SMU_833 led to extracellular DNA-dependent bacterial aggregation. In addition, the mutant was more susceptible to oxidative stress and less competitive against H_2_O_2_ producing oral streptococci. Quantitative proteomics analysis revealed that SMU_833 deficiency resulted in the significant downregulation of 10 proteins encoded by a biosynthetic gene cluster responsible for the production of mutanobactin, a compound produced by S. mutans which helps it survive oxidative stress. Tandem affinity purification demonstrated that SMU_833 interacts with the synthetic enzymes responsible for the production of mutanobactin. Similar to the *smu_833* mutant, the deletion of the mutanobactin gene cluster rendered the mutant less competitive against H_2_O_2_-producing streptococci. Our studies revealed a new link between SMU_833 virulence and mutanobactin, suggesting that SMU_833 represents a new virulent target that can be used to develop potential anticaries therapeutics.

**IMPORTANCE** Streptococcus mutans is the major bacterium associated with dental caries. In order to thrive on the highly populated tooth surface and cause disease, S. mutans must be able to protect itself from hydrogen peroxide-producing commensal bacteria and compete effectively against the neighboring microbes. S. mutans produces mutacins, small antimicrobial peptides which help control the population of competing bacterial species. In addition, S. mutans produces a peptide called mutanobactin, which offers S. mutans protection against oxidative stress. Here, we uncover a new link between the putative glycosyltransferase SMU_833 and the mutanobactin-synthesizing protein complex through quantitative proteomic analysis and a tandem-affinity protein purification scheme. Furthermore, we show that SMU_833 mediates bacterial sensitivity to oxidative stress and bacterial ability to compete with commensal streptococci. This study has revealed a previously unknown association between SMU_833 and mutanobactin and demonstrated the importance of SMU_833 in the fitness of S. mutans.

## Abstract (precis)

Streptococcus mutans is the major bacterium associated with dental caries. In order to thrive on the highly populated tooth surface and cause disease, S. mutans must be able to protect itself from hydrogen peroxide-producing commensal bacteria and compete effectively against the neighboring microbes. S. mutans produces mutacins, small antimicrobial peptides which help control the population of competing bacterial species. In addition, S. mutans produces a peptide called mutanobactin, which offers S. mutans protection against oxidative stress. Here, we uncover a new link between the putative glycosyltransferase SMU_833 and the mutanobactin-synthesizing protein complex through quantitative proteomic analysis and a tandem-affinity protein purification scheme. Furthermore, we show that SMU_833 mediates bacterial sensitivity to oxidative stress and bacterial ability to compete with commensal streptococci. This study has revealed a previously unknown association between SMU_833 and mutanobactin and demonstrated the importance of SMU_833 in the fitness of S. mutans.

## INTRODUCTION

Dental caries, or tooth decay, is characterized by the demineralization and destruction of tooth enamel. Streptococcus mutans is considered a major etiological agent of dental caries, especially in the initial formation of cavities and in the development of early childhood caries (1, 2). This is because of its ability to from a tenacious biofilm on the surfaces of teeth, produce copious amounts of acids through the fermentation of sugars, and survive the resulting low-pH environment (3, 4). The ability of S. mutans to cope with the acidic environment derives from its ability to maintain pH across the cell membrane, with the interior being more alkaline than the exterior. This is accomplished by altering the composition of membrane fatty acids and the production of its F_1_F_0_-ATPase pump (5–7). Furthermore, malolactic fermentation, part of the acid tolerance response in S. mutans, converts l-malate to l-lactate (a weaker acid than malate) and carbon dioxide, and regenerates ATP, which is needed for the transport of protons across the membrane (8). S. mutans also utilizes the enzyme carbonic anhydrase (Cah), which catalyzes the conversion of carbon dioxide to bicarbonate, helping to modulate pH (9).

S. mutans is one of hundreds of bacterial species that reside in the mouth and must compete with other bacteria to colonize and survive (10, 11). Commensal bacteria such as Streptococcus sanguinis and Streptococcus gordonii are considered early colonizers and typically colonize teeth prior to S. mutans (12). Commensal streptococci and mutans streptococci are known competitors. S. mutans produces mutacins to kill the commensals, and the commensals produce hydrogen peroxide to control S. mutans (13). Although S. mutans lacks catalase, it does contain several genes involved in defense against oxidative stress (*trxA* and *trxB*, thioredoxin reductase; *dpr*, peroxide resistance protein; *sod*, superoxide dismutase; *ahpC* and *ahpF*, alkyl hydroperoxide reductase C and F subunits; *gor*, glutathione reductase; *tpx*, thiol peroxidase; *nox-2*, water-forming NADH oxidase; and *spxA1* and *spxA2/spxB*, suppressors of clpP and clpX) (14, 15). In addition, S. mutans contains a genomic island, TnSmu2 (*smu_1334* to *smu_1349*), which harbors a nonribosomal peptide synthetase-polyketide synthase gene cluster. These genes encode proteins that are responsible for the production of mutanobactin, a compound involved in oxygen and hydrogen peroxide tolerance (16).

S. mutans also produces bacteriocins, peptide antibiotics that kill or inhibit the growth of closely related strains helping to compete against other bacteria. The bacteriocins of S. mutans are also called mutacins. S. mutans UA159 produces three nonlantibiotic mutacins: mutacins IV, V, and VI. Mutacin IV is encoded by two genes, *nlmA* and *nlmB*, whereas mutacins V and VI are encoded by *nlmC* and *nlmD*, respectively (17–19). The ComCDE, VicRK, and the more recently discovered HdrRM and BrsRM regulatory systems are responsible for controlling the expression of the mutacins (19–22).

The protein SMU_833 has been shown to be important for cariogenic biofilm formation and bacterial virulence in a rat caries model (23). Loss of the protein resulted in a decrease in glucosyltransferases (GtfB, GtfC, and GtfD), a reduction in glucan biofilm matrix, and an increase in lysis-independent extracellular DNA release (23). However, the effect of SMU_833 on the other virulence properties and overall bacterial fitness is unknown. *smu_833* resides just downstream of the main operon (*smu_825* to *smu_830*) responsible for the production of the rhamnose-glucose polysaccharides (RGPs) located on the cell surface. It was suggested that *smu_833* may play a role in their regulation, and it was given the name RgpI (24, 25). One study revealed that deletion of *rgpG* (*smu_246*), which results in defective RGP synthesis (26), had little effect on growth and stress tolerance but caused a defect in cell division and morphology, as well as biofilm formation (27). Conversely, another study done using the *rgpF* (*smu_830*) deletion mutant stated that deletion of *rgpF* resulted in cells being much more sensitive to various stressors and much less competitive *in vitro* and in an *in vivo* waxworm model (28). Differences in strains and experimental conditions can be attributed to phenotypic differences, but more work needs to be done to fully elucidate the contribution of the RGPs to the fitness and virulence of S. mutans.

In this study, we further investigated the function of SMU_833 and uncovered additional affected virulence factors. It was determined that resistance to oxidative stress was diminished in the *smu_833* mutant; however, the acid tolerance was not affected. Furthermore, the mutant lost the ability to inhibit oral commensal bacteria, likely via a defect in the synthesis of mutacin. Quantitative proteomics analysis uncovered 82 proteins that are significantly differentially expressed. Some specific targets may be directly affected by SMU_833. We demonstrate here, using tandem affinity purification (TAP), that SMU_833 interacts with the mutanobactin protein cluster and is likely needed for the production of the compound that confers resistance to oxidative stress. This study highlights a new link between SMU_833 and the oxidative stress-resistant compound and further demonstrates the importance of SMU_833 in the fitness and virulence of S. mutans.

## RESULTS

### Deletion of *smu_833* has no effect on growth rate but causes cells to aggregate.

**FIG 1:**
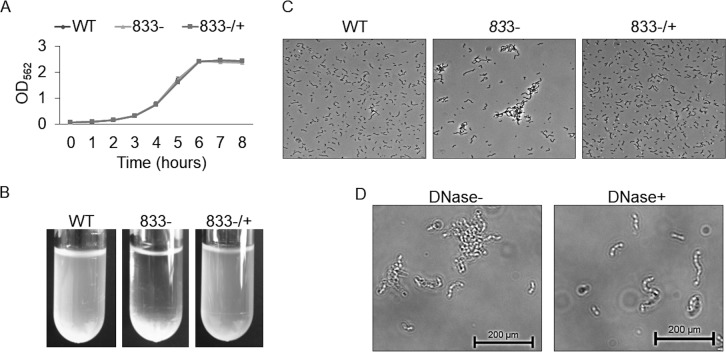
Effect of *smu_833* deletion on bacterial growth and aggregation. (A) Growth of wild-type, *smu_833* mutant, and complemented strains. Bacteria were grown statically in THB at 37°C with 5% CO_2_, and the OD_470_ was measured every hour for 8 h. (B and C) Visualization of cellular aggregation in the mutant with images of overnight cultures (B) and phase-contrast microscopy (C). (D) Phase-contrast microscopy after a 30-min DNase treatment of the mutant strain. The images are representative, but each was prepared in triplicate.

It was previously reported that SMU_833 in S. mutans is needed for the development of proper cariogenic biofilms and for both colonization and virulence in an *in vivo* rat model (23). However, its effects on other virulence factors and overall bacterial fitness are unknown. Deletion of *smu_833* has no effect on the bacterial growth rate (Fig. 1A). However, there was noticeable bacterial precipitation at the bottom of the culture tube when the *smu_833* mutant was grown overnight (Fig. 1B). Phase-contrast microscopy revealed that the mutant strain aggregates (Fig. 1C). Deletion of *smu_833* was previously shown to contain more extracellular DNA (eDNA) within the supernatant (23). To test whether the increase in eDNA is responsible for the cellular aggregation, we incubated the mutant strain with DNase and inspected the cells using phase-contrast microscopy again. When incubated with DNase, the cells of the mutant strain no longer aggregated (Fig. 1D), indicating that the aggregation is related to the increase in eDNA.

### Deletion of *smu_833* increased susceptibility to oxidative stress but not low pH.

**FIG 2:**
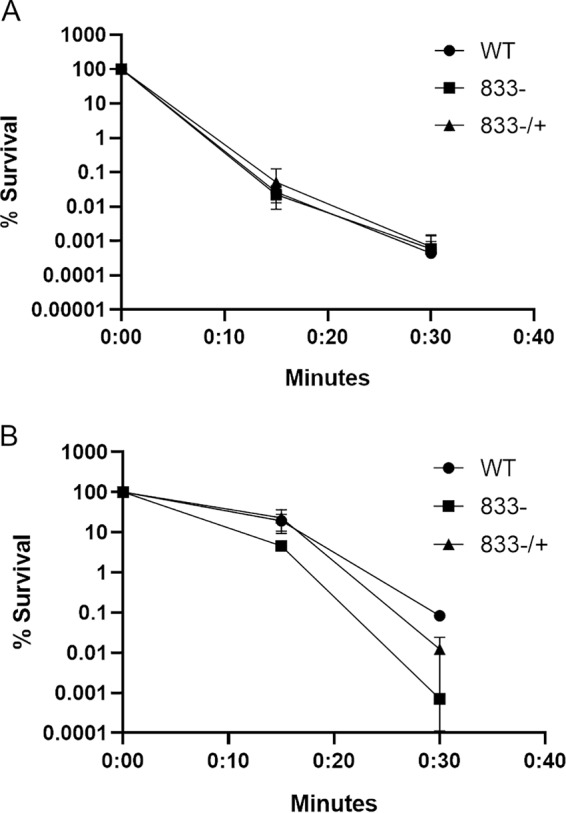
Loss of SMU_833 did not affect acid tolerance but reduced the ability to resist oxidative stress. Wild-type (WT), *smu_833* mutant (833–), and complemented (833–/+) strains were grown to early exponential phase, washed with 0.1 M glycine (pH 7.0), and then resuspended in either 0.1 M glycine buffer (pH 2.8) for acid killing (A) or 0.1 M glycine buffer (pH 7.0) with 0.2% hydrogen peroxide (B). The data are averages of three independent experiments. Error bars represent the standard deviations, but are not depicted if the value was 0 or a negative number. Statistical analysis was performed using a Student *t* test to compare the means of two samples. Comparison of WT and mutant (833–) strains: 15 min, *P* < 0.05; 30 min, *P* < 0.01.

Through fermentation of sugars, S. mutans, as well as other bacteria, creates a very acidic environment on the surfaces of teeth. The ability to establish a low-pH environment, acidogenicity, and the ability to survive exposure to a low-pH environment, aciduricity, are virulence traits of S. mutans. Our recent study shows that the *smu_833* mutant does not form a biofilm that is as acidic as the wild type (WT) (23). To determine how well the mutant can survive a low pH compared to the wild-type strain, we exposed the strains to pH 2.8 and evaluated their survival (Fig. 2A). The *smu_833* mutant showed no defect in its ability to survive exposure to pH 2.8 compared to the wild type (Fig. 2A).

There are hundreds of bacterial species present in the mouth (10, 11). Many of the early colonizers of tooth surfaces are hydrogen peroxide-producing commensal species such as Streptococcus sanguinis and Streptococcus gordonii (13). The hydrogen peroxide produced by these commensals is one of the factors they employ to keep cariogenic bacteria such as S. mutans at bay. Therefore, we wanted to test whether the deletion of *smu_833* affects the ability *of* S. mutans to survive exposure to hydrogen peroxide. After 15 min of exposure to hydrogen peroxide, the mutant showed a slight defect in the ability to survive (Fig. 2B). However, by 30 min, there was an ∼2-log decrease in the ability of the *smu_833* mutant to survive (Fig. 2B). These data demonstrate that SMU_833 is important for oxidative stress tolerance but not for low-pH stress.

### Loss of SMU_833 decreases competitive fitness of *S. mutans* and results in decreased mutacin gene expression.

**FIG 3:**
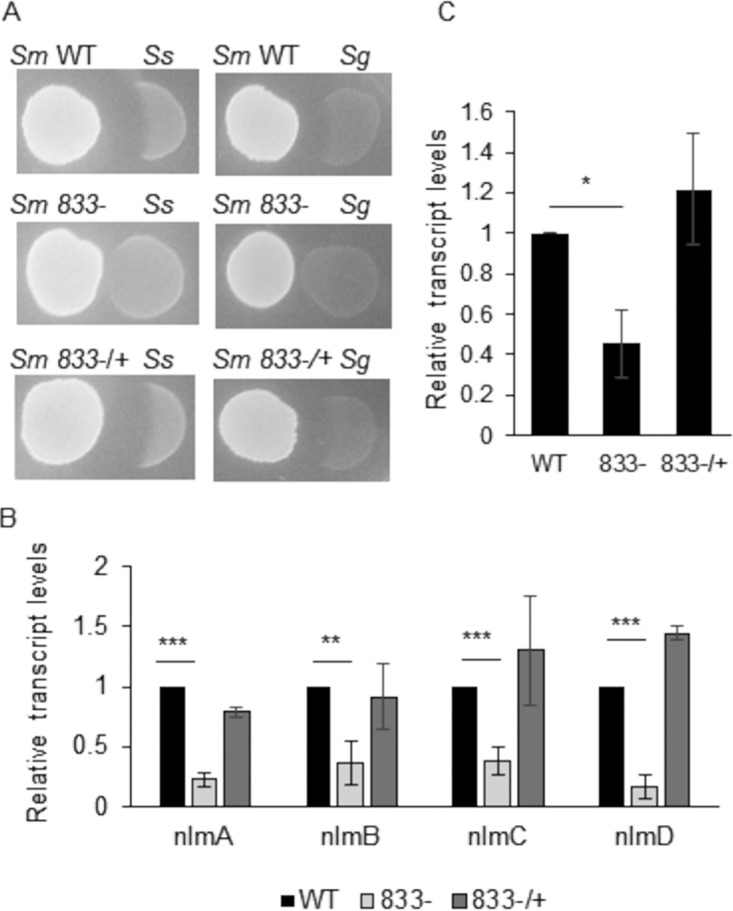
Loss of SMU_833 decreased bacterial competition and mutacin expression. (A) Plate competition assay of S. mutans (*Sm*) wild-type, *smu_833* mutant, and complemented strains with S. sanguinis (*Ss*) and S. gordonii (*Sg*). S. mutans strains were the initial colonizers plated for 24 h prior to the secondary colonizers. The images are representative of at least three independent experiments. (B and C) Expressions of the *nlmA*, *nlmB*, *nlmC*, and *nlmD* (B) and *comE* (C) genes were measured in wild-type, *smu_833* mutant, and complementation strains using qRT-PCR. The data are averages from a representative experiment performed at least three times. Statistical analysis was performed using a Student *t* test (***, *P* < 0.001; **, *P* < 0.01; *, *P* < 0.05).

One of the strategies S. mutans utilizes to outcompete oral commensal bacteria is the production and secretion of mutacins. We wanted to test the ability of our *smu_833* mutant strain to compete with the oral commensals S. sanguinis and S. gordonii. The *smu_833* mutant lost the ability to inhibit the oral commensal bacteria, whereas the wild type and its complement effectively competed with commensal streptococci (Fig. 3A). To determine whether the defect may be related to the production or to the secretion of mutacins, quantitative reverse transcription-PCR (qRT-PCR) was performed to determine the transcript levels of the four mutacin genes: *nlmA*, *nlmB*, *nlmC*, and *nlmD*. There was a significant decrease in the levels of all four genes in the *smu_833* mutant (Fig. 3B), suggesting the reduced competitiveness of the mutant is at least partly due to a decrease in the transcription of mutacins. Mutacin levels are also known to be affected and regulated by two-component systems (19–21). The levels of *comE*, a response regulator known to regulate mutacin production, were reduced significantly in the *smu_833* mutant strain (Fig. 3C). Though the reduction in *comE* is not as dramatic as the reduction in mutacin genes, it may also partially explain the decrease in the transcription of mutacin genes.

### Quantitative proteomics analysis of the *smu_833* mutant.

**FIG 4:**
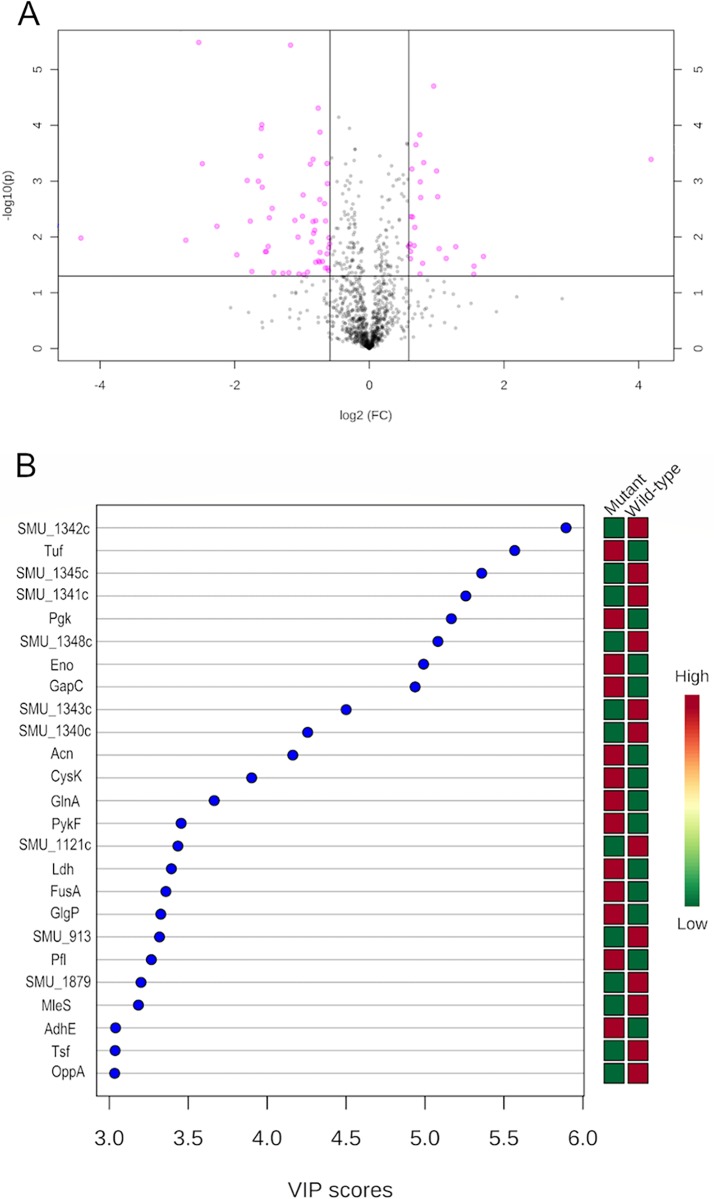
Quantitative SWATH-MS proteomics revealed significant decrease in expression of the mutanobactin synthesizing complex in the *smu_833* mutant. (A) Important features selected by a volcano plot with a fold change (FC) threshold (*x*) 1.5 and a *t* test threshold (*y*) of 0.05. The pink circles represent features above the threshold. (B) VIP plot. The graph displays the top 25 most important proteins identified by PLS-DA. These proteins contribute the most to the variance. Colored boxes on right indicate the relative concentrations of corresponding proteins for samples from the wild-type and *smu_833* mutant strains. VIP is a weighted sum of squares of the PLS-DA loadings, taking into account the amount of explained Y-variable in each dimension.

We wanted to gain insight on the role *smu_833* plays globally in S. mutans to better understand how SMU_833 affects biofilm, eDNA release, oxidative stress resistance, and mutacin production. To do this, we performed SWATH-MS quantitative proteomics analysis (29) on lysate samples from wild-type and *smu_833* mutant strains. S. mutans UA159 contains 1,963 open reading frames (30) and the proteomics analysis was able to detect 1,042 proteins. Univariate analyses of the data were performed using MetaboAnalyst 4.0 (31). The volcano plot illustrates significant changes in the *smu_833* mutant (Fig. 4A). There are 82 proteins depicted as pink circles above the designated cutoffs (fold change, >1.5; *P* < 0.05). The majority (56/82) of these proteins were found to be decreased in the *smu_833* mutant; however, many were described either as uncharacterized or only as having a putative function. The most interesting finding from our quantitative proteomics study was the discovery that 10 proteins from a single operon responsible for the synthesis of mutanobactin were decreased (Table 1). Nine of the 10 were decreased by >2-fold. Mutants lacking mutanobactin have been shown to be more sensitive to oxidative stress (16), a phenotype shared with the *smu_833* mutant. In addition, there were two cell surface proteins, WapA and GbpC, that decreased moderately (2- to 3-fold) but very significantly (*P* < 0.005). There is a decrease in three putative transcription regulators, and one of them, SMU_1287, was the most decreased protein in the mutant. Carbonic anhydrase Cah and, to a greater extent, malolactic enzyme MleS, two proteins involved in acid stress tolerance, were also found to be decreased in the mutant.

**TABLE 1:** Proteins decreased in the *smu_833* mutant

Putative function and locus	Description	Fold change	*P*
Mutanobactin cluster			
SMU_1348c	Putative ABC transporter ATP-binding protein	0.1728	3.28E−06
SMU_1344c	Putative malonyl-CoA acyl-carrier-protein transacylase	0.3308	9.73E−05
SMU_1346	Putative thioesterase BacT	0.2845	0.000976
SMU_1343c	Putative polyketide synthase	0.3321	0.001288
SMU_1341c	Putative gramicidin S synthetase	0.3574	0.004544
SMU_1340	Putative surfactin synthetase BacA2	0.3529	0.014805
SMU_1345c	Putative peptide synthetase	0.3466	0.018274
SMU_1342	Putative bacitracin synthetase 1 BacA1	0.3428	0.018378
SMU_1349	Uncharacterized protein	0.6021	0.018878
SMU_1347c	Uncharacterized protein	0.2984	0.041588
			
Cell wall proteins			
SMU_1396	Glucan-binding protein C, GbpC	0.3290	0.000114
SMU_987	Wall-associated protein WapA	0.5447	0.000497
			
Transcriptional regulators			
SMU_1287	Putative transcriptional regulator	0.0514	0.010463
SMU_1361c	Putative transcriptional regulator (TetR family)	0.1512	0.011459
SMU_61	Putative transcriptional regulator	0.5602	0.0053064
			
Acid tolerance			
SMU_137	Malolactic enzyme MleS	0.179	0.000488
SMU_1595	Putative carbonic anhydrase Cah	0.5058	0.001774

Of the 82 proteins, 26 were increased in the *smu_833* mutant, and only 8 of these 26 were increased ≥2-fold. The proteins of interest are categorized and listed in Table 2. There was an increase in three proteins (MsmK, MsmE, and GtfA) encoded by the *smu_876* to *smu_883* operon, which is responsible for the uptake and metabolism of melibiose, raffinose, and isomaltotriose and for the metabolism of sucrose (32); however, none of these reached a 2-fold increase. There was also an increase in three proteins involved in DNA replication and cell division. Thioredoxin (TrxA), a protein involved in oxidative stress resistance, was only modestly increased (1.5-fold) although significantly (0.015). The surface protease HtrA was increased about 2-fold, suggesting cell surface-related stress. Lastly, there was a drastic increase in an uncharacterized protein, SMU_739c, that has no known function.

**TABLE 2:** Proteins increased in the *smu_833* mutant

Putative function and locus	Description	Fold change	*P*
MSM operon			
SMU_882	Multiple sugar-binding transport ATP-binding protein MsmK	1.68	0.000148
SMU_878	Multiple sugar-binding protein MsmE	1.69	0.001033
SMU_881	Sucrose phosphorylase GtfA	1.52	0.013336
			
DNA replication/cell division			
SMU_1967	Single-stranded DNA-binding protein Ssb2	1.60	0.006748
SMU_20	Cell shape-determining protein MreC	2.02	0.001907
SMU_2165	Putative SpoJ, chromosome segregation protein	1.75	0.000467
			
Oxidative stress resistance			
SMU_1869	Thioredoxin TrxA	1.50	0.014973
			
Other			
SMU_2164	Serine protease HtrA	1.94	1.99E−05
SMU_739c	Uncharacterized protein	18.16	0.000408

Because of the high dimensionality of proteomics data, multivariate statistical analysis is well suited for determining statistically significant changes that arise from alterations created by the deletion of *smu_833*. Data were normalized and subjected to principal-component analysis (PCA) to explain variances in data without referring to class labels. Wild-type and mutant groups show very clear separation, and these two PCs can account for about 90% of the variation between the WT and the *smu_833* mutant (see Fig. S1 in the supplemental material). The normalized protein concentrations were also subjected to partial-least-squares–discriminant analysis (PLS-DA), a technique for identifying features that best describe the differences between groups. A variable importance in projection (VIP) score was calculated and used to examine the contribution of each variable to the variance. The top 25 variables or proteins identified by VIP scores are shown (Fig. 4B), and 6 of the top 10 contributing proteins belong to the mutanobactin locus. Taken together, both the univariate and the multivariate statistical analyses strongly suggest that there is a considerable connection between the deletion of *smu_833* and the decrease in mutanobactin proteins.

10.1128/mSphere.00429-19.1 FIG S1 Principal-component analysis (PCA) scores plot. The explained variance of each component is shown in brackets. Download FIG S1, TIF file, 0.3 MB. Copyright © 2019 Rainey et al. 2019 Rainey et al. This content is distributed under the terms of the Creative Commons Attribution 4.0 International license.

### SMU_833 is associated with the mutanobactin-producing enzyme complex.

**FIG 5:**
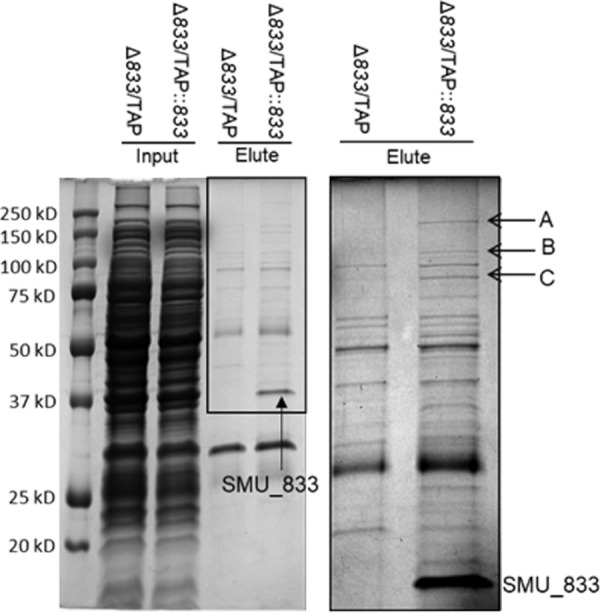
SMU_833 associates with mutanobactin synthetic enzymes determined by TAP. A Coomassie blue-stained SDS-PAGE gel of input and eluted samples from TAP purification is shown. The *smu_833* mutant with an empty TAP vector (*833-*/TAP) was used as a negative control. On the right is an enlargement of the eluted samples in the boxed area on the left. Bands A to C and the band labeled SMU_833 were identified by mass spectrophotometry. Bands A, B, and C were identified as SMU_1342, SMU_1340, and SMU_1341, respectively.

Proteomics studies revealed many different pathways SMU_833 may affect that give rise to the observed phenotypes. To further determine which proteins or pathways SMU_833 may more directly interact with, we performed tandem affinity purification (TAP), which utilizes two affinity purification steps to ascertain SMU_833 interacting partners. The elution fractions from SMU_833 TAP and its control were compared (Fig. 5). Bands evident in the SMU_833 TAP and absent in the negative control were subjected to liquid chromatography-mass spectrometry analysis. Bands A, B, and C were identified as SMU_1342, SMU_1340, and SMU_1341, respectively, and all three are part of the mutanobactin-synthesizing complex. Concomitantly, proteomics analysis showed a decrease in protein levels in either 6 (VIP plot) or 10 (volcano plot) proteins within the same mutanobactin operon. These findings provide strong evidence that SMU_833 interacts with proteins responsible for the production of mutanobactin, a compound produced by S. mutans which helps the bacteria survive exposure to oxidative stress.

**FIG 6:**
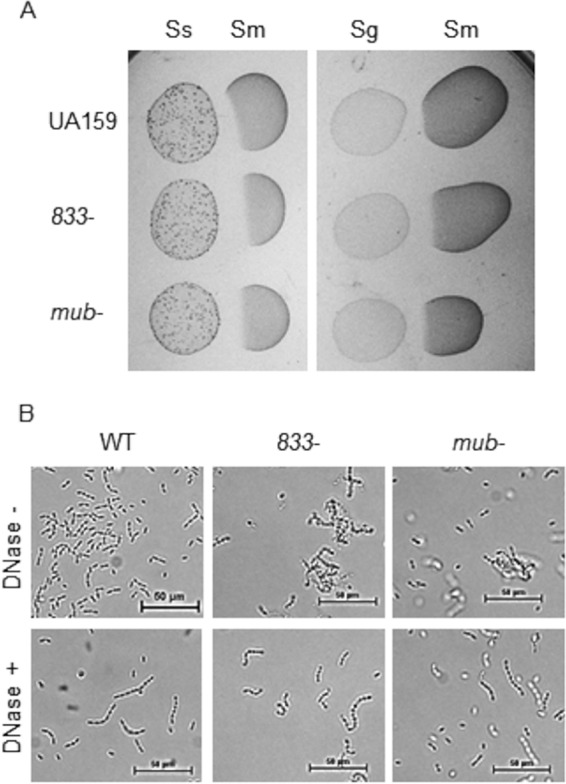
The mutanobactin and *smu_833* mutants exhibit similar phenotypes. (A) Plate competition assay of S. mutans (*Sm*) wild-type, *smu_833* mutant, and complemented strains with S. sanguinis (*Ss*) and S. gordonii (*Sg*). S. sanguinis and S. gordonii were used as the initial colonizers, and S. mutans strains were used as the secondary colonizers. (B) Visualization of cellular aggregation using phase-contrast microscopy after a 30-min DNase treatment. The images are representative but were determined in triplicate.

We showed that the *smu_833* mutant was also sensitive to oxidative stress (Fig. 2B). To further compare both the *smu_833* and the mutanobactin mutants side by side and to determine how the mutants would fare against exposure to commensals, we performed a plate competition assay wherein the commensals and the S. mutans strains were plated sequentially on the same plate (Fig. 6A). Both S. sanguinis and S. gordonii inhibited the mutanobactin and *smu_833* mutants more than they inhibited WT S. mutans, suggesting that the deletion of *smu_833* and mutanobactin exhibits a similar defect in competing with hydrogen peroxide-producing commensals.

The *smu_833* mutant exhibits DNA-dependent aggregation (Fig. 1C and D). Mutanobactin is a large complex within the membrane of S. mutans, so it is possible that loss of this complex could affect DNA release and cell aggregation. To test this, we also examined bacterial cells with or without incubation with DNase under phase-contrast microscopy. The mutanobactin mutant readily formed DNA-dependent cellular aggregates (Fig. 6B), a phenotype similar to the *smu_833* mutant, suggesting a connection between SMU_833 and the mutanobactin cluster.

## DISCUSSION

We have previously reported that SMU_833 is important for glucosyltransferase production, glucan matrix formation, and *in vivo* virulence in a rat model (23). However, its effects on overall bacterial fitness and other virulence factors remained unknown. The human mouth harbors hundreds of different bacterial species, and these species both cooperate and compete with one another (10, 11). Many of the initial colonizers are commensal bacteria such as Streptococcus sanguinis and Streptococcus gordonii, and they colonize the mouth prior to S. mutans (12). Commensal bacteria that produce hydrogen peroxide create oxidative stress conditions that are able to control S. mutans, and S. mutans that produces mutacins is able to limit the commensals (13); therefore, the ability to both produce mutacins and survive exposure to hydrogen peroxide are imperative for the survival and fitness of S. mutans.

Our studies show the *smu_833* mutant is more sensitive to inhibition by both commensals and their metabolite, hydrogen peroxide. The mutanobactin compound is important for oxygen and hydrogen peroxide tolerance (16). SMU_833 appears to directly interact with multiple components of the mutanobactin-synthesizing protein complex (Fig. 5), and the levels of 10 proteins in this complex significantly decreased when *smu_833* was inactivated (Table 1). SMU_833 has two predicted transmembrane helices and therefore is likely localized to the cell membrane, where the mutanobactin protein complex also resides. As a putative glycosyltransferase, it is possible that SMU_833 is needed for the stability or regulation of the complex through their interactions. The SMU_833 homologue PgfS has been shown to glycosylate the collagen-binding protein, Cnm, which renders Cnm more stable (33). Numerous other studies have demonstrated the importance of protein-protein interactions to the function, solubility, and stability of protein complexes (34–38). Additional investigation is required to validate biological outcomes of this type of interaction between SMU_833 and the mutanobactin-synthesizing complex.

S. mutans lacks catalase, but it does contain numerous other proteins that help it to survive oxidative stress conditions (14, 15). As noted in Table 2, there was an increase in TrxA, one of the proteins involved in oxidative stress resistance in S. mutans. This supports the finding that the deletion of *smu_833* causes the cell to become more sensitive to oxidative stress or that the mutant experiences more severe oxidative stress. Bacterial cells used for the proteomic studies were not preexposed to oxidative stress. It is possible we would observe greater differences in the production of oxidative stress resistance gene products between the wild-type and mutant strains if they were challenged with oxidative stress conditions. The sensitivity of the *smu_833* mutant to hydrogen peroxide (Fig. 2B) and commensals (Fig. 6A), along with decreased mutacin production (Fig. 3B) and losing the ability to inhibit commensals (Fig. 3A), helps to explain why the *smu_833* mutant, though able to form an abundant biofilm, was unable to maintain robust colonization and be as cariogenic as the wild-type bacteria on rat tooth surfaces (23). Another recent study revealed that SMU_833 is needed for optimal fitness in minimal medium and for colonization in a different animal model, but it is not as important for fitness in rich medium (39). Our unpublished observations support this and confirm a growth defect for the *smu_833* mutant in minimal media. Most mutations in S. mutans that render the bacteria more sensitive to oxidative stress also result in the bacteria being more sensitive to acidic stress. Therefore, it is noteworthy that SMU_833 appears to be important in nutrient-limiting, oxidative-stress, and highly competitive conditions but not in low pH. The proteomics analysis revealed no changes in the levels of F-ATPase components needed for the removal of protons from inside the cell (7) but decreases in carbonic anhydrase (Cah) and malolactic enzyme (MleS), also known to play a role in acid tolerance (8, 9). However, the *smu_833* mutant does contain increased levels of cell-associated eDNA, leading to cellular aggregation. It is possible that the increase in negatively charged eDNA and aggregation act as a barrier to shield the mutant from low-pH stress.

The loss of SMU_833 did not impact the growth of the bacterium, but it did induce bacterial aggregation that was reversed after treatment with DNase. Increased lysis-independent DNA release was previously reported in the *smu_833* mutant (23), and the increased DNA-dependent aggregation is likely linked to this finding. In fact, proteomics revealed increases in three cell division proteins—the cell shape-determining protein MreC, the chromosomal segregation protein SpoJ, and the single-stranded DNA-binding protein Ssb2—in the mutant (Table 2), which is relevant to DNA synthesis. One potential action of lysis-independent DNA release in Gram-positive bacteria takes place during cell division. In Enterococcus faecalis, eDNA is localized at the septum, and the release is believed to occur from metabolically active, living cells with membrane potential (40). MreC, a protein involved in peptidoglycan biosynthesis, is also localized at the septum in Streptococcus pneumoniae (41), suggesting a possible connection and explanation for increased eDNA release in the *smu_833* mutant. SpoJ is involved in chromosome segregation and condensation, and deletion of SpoJ (ParB) resulted in an increase in cells containing little to no DNA (42). In addition, Ssb2 is important for DNA replication and repair, and it is able to bind single-stranded DNA and an array of partner proteins to recruit them to their sites of action (43). Together, the increase in these proteins may result in increased DNA replication and release more eDNA from the *smu_833* mutant cells.

Furthermore, we also determined that the loss of SMU_833 severely decreased the ability of S. mutans to inhibit the oral commensals S. sanguinis and S. gordonii, indicating that the production and/or secretion of mutacins is diminished (Fig. 3A). qRT-PCR analysis showed that the expression levels of all four mutacin genes (*nlmA*, *nlmB*, *nlmC*, and *nlmD*) had decreased significantly (Fig. 3B), which suggests that the synthesis of mutacins is limited. The levels of *comE*, the major known regulator of mutacin gene expression (19), were also decreased in the mutant, but to a much lesser extent than the mutacin genes themselves (Fig. 3B). ComE plays an additional role in quorum sensing and competence development (44). A recent study found that *smu_833* mutant to have decreased transformation efficiency, as well as exhibiting less inhibition against S. gordonii (45). It was suggested that SMU_833 may impact cell membrane-associated sensing and signal transduction systems. It is possible that SMU_833 exerts its effects on bacterial physiology and cellular signaling systems via its association with the mutanobactin complex in addition to its suggested impact on RGP synthesis. A less severe decrease in *comE* compared to the mutacin genes could also indicate there is a second regulator of mutacin expression altered within the *smu_833* mutant. Whether additional regulators of mutacin production are involved or modulated remains elusive, even with the proteomics data. The other known regulators, HdrRM and BrsRM, responsible for controlling the expression of the mutacins (20, 21) were not detected by our proteomics analysis. Furthermore, a screen of all response regulators of S. mutans was performed with qPCR (data not shown), and there was no change in the transcript levels for any of the response regulators with a known or putative function in the synthesis of mutacins found in UA159 (46).

This study revealed that loss of SMU_833 hinders the ability of S. mutans to survive and compete in the oral cavity by severely reducing mutacin expression, eliminating its ability to inhibit oral commensals, and making the mutant more sensitive to commensal bacteria and their by-product, hydrogen peroxide. The proteomics study revealed that loss of SMU_833 resulted in a significant decrease in numerous proteins involved in the synthesis of mutanobactin. Furthermore, SMU_833 associated with the mutanobactin biosynthetic complex and mutants of each exhibited similar oxidative stress and DNA-dependent aggregation phenotypes. This connection provides an explanation for some of the phenotypes displayed by the *smu_833* mutant. Lastly, proteomics uncovered new leads, which need to be studied further to fully uncover the function of SMU_833 in S. mutans.

## MATERIALS AND METHODS

### Bacterial strains and culture conditions.

Streptococcus mutans UA159, Streptococcus sanguinis SK36, Streptococcus gordonii DL1, and Escherichia coli Top10 were used in this study. The *smu_833* mutant and its complement were made previously from the UA159 background (23). The mutanobactin (*mub*) deletion mutant was obtained from Fengxia Qi at the University of Oklahoma Health Science Center (16). Streptococci were maintained in Todd-Hewitt broth (THB) or TH agar and grown statically at 37°C with 5% CO_2_. E. coli strains were grown in Luria-Bertani (LB) broth or on LB agar plates and grown aerobically at 37°C. Antibiotics were used at the following concentrations: 1 mg ml^−1^ kanamycin and 10 μg ml^−1^ erythromycin for S. mutans, and 50 μg ml^−1^ kanamycin and 300 μg ml^−1^ erythromycin for E. coli.

### Growth curve and phase-contrast microscopy.

For growth studies, bacterial strains were grown statically in THB at 37°C with 5% CO_2_. Overnight cultures were diluted into fresh media, and the optical density at 470 nm (OD_470_) was measured every hour for 8 h. Phase-contrast microscopy was performed by placing 10 μl of vortexed planktonic culture on a microscope slide and using the 60× objective magnification on a Nikon Eclipse TE2000-E inverted microscope. DNase treatments were performed for 30 min in a 37°C water bath using RQ1 RNase-free DNase (Promega). At least three independent experiments were performed, and the images displayed are representative.

### Acid and hydrogen peroxide killing.

The ability of bacterial strains to withstand acid and hydrogen peroxide challenge was determined using protocols described elsewhere (47, 48). Briefly, overnight cultures of S. mutans strains were diluted and grown to an OD_470_ of 0.5. Cultures were centrifuged, the supernatant was discarded, and the cells were resuspended in 1/3 original volume 0.1 M glycine buffer (pH 7.0) to wash them. The cells were centrifuged again and resuspended in either 0.1 M glycine buffer (pH 2.8) for acid killing, 0.1 M glycine buffer (pH 7.0) with 0.2% hydrogen peroxide, or 0.1 M glycine buffer (pH 7.0) as a control. Aliquots were taken at 0, 15, and 30 min and then diluted and plated on TH agar plates for enumeration of the CFU.

### Competition assay on solid medium.

The ability of S. mutans or oral commensal bacteria (S. sanguinis or S. gordonii) to inhibit the other on solid agar was assessed as described previously (49, 50). Briefly, a 10-μl subculture of the initial colonizer was inoculated onto a TH agar plate and grown overnight at 37°C with 5% CO_2_. After the overnight incubation, 10 μl of subcultured competing strain was inoculated next into the initial colonizer and grown overnight at 37°C with 5 % CO_2_. Growth inhibition was evaluated by the presence of a zone of inhibition where the two bacteria intersect.

### Quantitative real-time PCR.

RNA was extracted from exponential-phase-grown S. mutans cultures using a Direct-Zol kit (Zymo Research), and DNA was digested using RQ1 RNase-free DNase (Promega). RNA was then purified with an RNeasy Minikit (Qiagen) and converted into cDNA using an iScript cDNA synthesis kit (Bio-Rad). cDNA was then used for qRT-PCR with iQ SYBR green Supermix (Bio-Rad). The primers used to amplify 16S rRNA and the tested genes are presented in Table 3.

**TABLE 3:** Primers used in this study

Primer	DNA sequence (5′–3′)	Description
16S rRNA forward	CTTACCAGGTCTTGACATCCC	qRT-PCR
16S rRNA reverse	CCAACATCTCACGACACGAG	qRT-PCR
*nlmA* forward	ATGGATACACAGGCATTTC	qRT-PCR
*nlmA* reverse	TATGGGGTAACAAGAGTCC	qRT-PCR
*nlmB* forward	TGTCAGAAGTTTTTGGTGGA	qRT-PCR
*nlmB* reverse	AGCACATCCAGCAAGAATA	qRT-PCR
*nlmC* forward	AGCATATGGACCAAGAAATG	qRT-PCR
*nlmC* reverse	ACGTAATGGATAATGAAGCAC	qRT-PCR
*nlmD* forward	GGGTGGTGGTATGATTAG	qRT-PCR
*nlmD* reverse	AACGACTGGGAGAGTAAC	qRT-PCR
*comE* forward	TCATACTGCCGTAGAATTCA	qRT-PCR
*comE* reverse	AAGAATGGTCAATCAGAGGA	qRT-PCR
*smu_833* SalI forward	GACCGTGTCGACAATGAAAAAACTTTCAATAG	pVPT-TAP::*smu_833*
*smu_833* KpnI reverse	GGTCAGGGTACCTCCTTTTTCCTTAAC	pVPT-TAP::*smu_833*

### Protein sample processing for mass spectrometry.

Protein bands were excised from the gel and disulfide bonds were reduced using dithiothreitol (25 mM) at 50°C for 30 min, followed by alkylation of free thiols groups with iodoacetamide (55 mM) for 30 min in the dark at room temperature. After removal of excess alkylating agent, the gel pieces were evaporated to dryness prior to reswelling in 100 mM ammonium bicarbonate buffer and overnight digestion using Promega Gold trypsin (12.5 ng/ml). Tryptic peptides were extracted using solution of 1% formic acid in water and acetonitrile (50/50) and then evaporated to dryness in a SpeedVac. Samples were resuspended in 50 μl of double-distilled water (ddH_2_O)–0.1% formic acid for mass spectrometry evaluation.

### Generation of *Streptococcus mutans* protein library.

An aliquot (5 μl) of each digest was loaded onto a Nano cHiPLC 200 μm ID × 0.5 mm ChromXP C_18_-CL 3-μm 120-Å reverse-phase trap cartridge (Eksigent, Dublin, CA) at 2 μl/min using an Eksigent 415 LC pump and autosampler. After the cartridge was washed for 10 min with 0.1% formic acid in ddH_2_O, the bound peptides were flushed onto a Nano cHiPLC 200-μm ID × 15-cm ChromXP C_18_-CL 3-μm 120-Å reverse-phase column (Eksigent) with a 100-min linear (5 to 50%) acetonitrile gradient in 0.1% formic acid at 1,000 nl/min. The column was then washed with 90% acetonitrile–0.1% formic acid for 5 min and reequilibrated with 5% acetonitrile–0.1% formic acid for 15 min. A Sciex 5600 Triple-TOF mass spectrometer (Sciex, Toronto, Canada) was used to analyze the protein digest. The IonSpray voltage was 2,300 V, and the declustering potential was 80 V. Ion spray and curtain gases were set at 10 and 25 lb/in^2^, respectively. The interface heater temperature was 120°C. Eluted peptides were subjected to a time-of-flight survey scan from *m/z* 400 to 1250 to determine the top 20 most intense ions for tandem mass spectrometry (MS/MS) analysis. Product ion time-of-flight scans (50 ms) were carried out to obtain the MS/MS spectra of the selected parent ions over the range from *m/z* 400 to 1,000. The spectra were centroided and deisotoped by Analyst software (v1.7 TF; Sciex). A β-galactosidase trypsin digest was used to establish and confirm the mass accuracy of the mass spectrometer.

The MS/MS data were processed to provide protein identifications using an in-house Protein Pilot 4.5 search engine (Sciex) using the UniProt Streptococcus mutans protein database and a trypsin digestion parameter and carbamidomethylation for alkylated cysteines as a fixed modification. Proteins of significance were accepted based on the criteria of having at least two peptides detected with a confidence score of ≥95% using the Paradigm method embedded in the Protein Pilot software.

### Quantitative SWATH-MS.

The same chromatographic conditions described for the building of the S. mutans Protein Library were used for SWATH-MS (sequential windowed acquisition of all theoretical fragment ion mass spectra) analysis. Eluted peptides were subjected to SWATH-MS. In each 1.25-ms duty cycle, a high-resolution TOF MS1 scan (250 ms) was obtained, followed by 40 sequential product ion scans (50 ms) with 25 *m/z* windows starting at *m/z* 400 through *m/z* 1,000. These were collected using a collision energy of 35 eV and a collision energy spread of 15 eV. The spectra are centroided and deisotoped by Analyst software (v1.6 TF; Sciex). A β-galactosidase trypsin digest was used to establish and confirm the mass accuracy of the mass spectrometer.

The MS/MS data were deconvoluted and processed using PeakView software 1.2 with the SWATH application (Sciex). The software uses the pregenerated protein identification library to construct individual peptide signatures to compare with peptides in individual samples after analysis. This data-independent approach allows the collection of sufficient data as a peak elutes from the column to determine the area under the peak. In addition, all of the peptides that eluted from the column were subjected to MS/MS analysis, thereby creating a complete digital library for the sample.

### Univariate and multivariate analysis of proteomics data using MetaboAnalyst 4.0.

The areas of the tryptic peptides for each protein were added together. Two-column Excel .csv files were then created for each sample, containing on each line a numeric code for the protein and the summed tryptic peak areas. The samples were then placed into two folders for wild-type and mutant S. mutans strains. The folders were combined into a .zip file and submitted to MetaboAnalyst 4.0 (31) for univariate (Volcano plot) and multivariate (PCA and PLS-DA) statistical analyses. VIP scores using component 1 that were >3.0 were considered significant. The data that support the findings of this study are available from the corresponding author upon reasonable request.

### Tandem affinity purification.

PCR amplification of *smu_833* from S. mutans UA159 using the primers listed in Table 3 was used for the construction of the pVPT-*tap-smu_833* plasmid. DNA was digested with SalI and KpnI and ligated into the pVPT-*tap* vector. This resulted in the creation of vector containing *smu_833* attached to a TAP tag which consists of two IgG binding domains of Staphylococcus aureus protein A (ProtA) and a calmodulin-binding peptide separated by a protease cleavage site (51). IgG and calmodulin beads were then used to double affinity purify SMU_833 and any interacting partners. TAP was performed as described previously (36, 51, 52) with modifications. Briefly, 1-liter bacterial cultures were grown to an OD_470_ of 1.0, harvested, and frozen at −80°C. The pellets were thawed and resuspended in 10 ml of NETN (100 mM NaCl, 20 mM Tris-HCl [pH 8.0], 0.5 mM EDTA, 0.5% [vol/vol] NP-40). Glass beads were added, and cells were lysed by vortexing with the beads for 1 h. The lysates were centrifuged, and the supernatant was transferred to a new tube for purification, which was carried out as described previously (36, 51) except with NETN buffer. Both the whole-cell lysates and the elution fractions were run on an SDS-PAGE gel. The negative control was the *smu_833* mutant with the empty pVPT-TAP vector and was used as a comparison to the *smu_833* mutant with the pVPT-TAP-*smu_833* vector. The protein bands present in the test strain and absent in the negative control were considered bands of interest and subjected to mass spectrophotometry analysis.
