# Supplementary figures and images for: Quantitative Proteomics Uncovers the Interaction between a Virulence Factor and Mutanobactin Synthetases in Streptococcus mutans
Source: mSphere. 2019 Sep 25;4(5):e00429-19. doi: 10.1128/mSphere.00429-19 (PMC6763767; doi:10.1128/mSphere.00429-19)

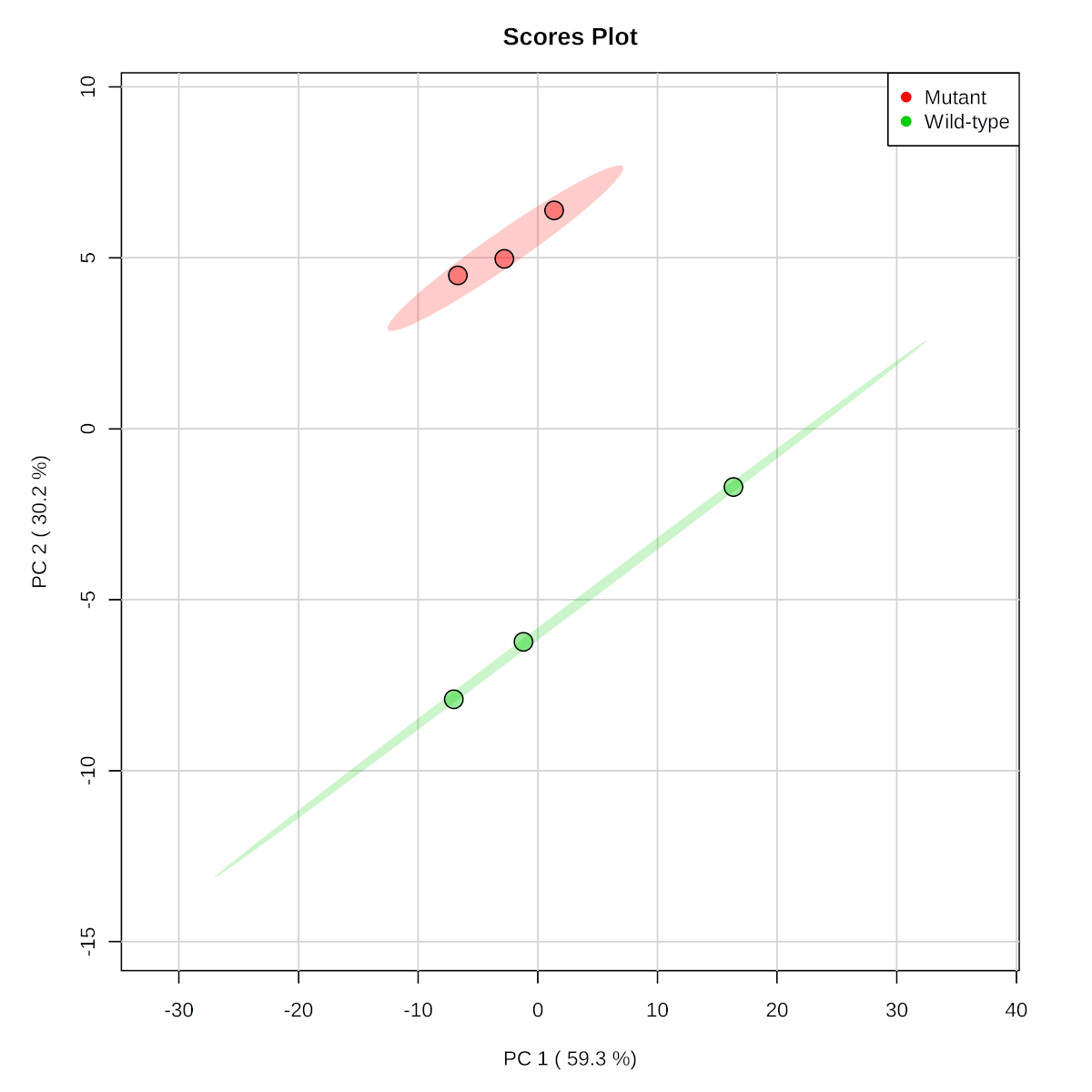

Supplement: FIG S1 [file mSphere.00429-19-sf001.tif]
